# Evaluation of the Necessity of Repeat Biopsy in Patients with Thyroid Nodules Classified as Atypia of Undetermined Significance (AUS) Based on Fine-Needle Aspiration Biopsy Results

**DOI:** 10.3390/medicina61071196

**Published:** 2025-06-30

**Authors:** Yiğit Türk, Bahadır Emre Baki, Özer Makay, Gökhan İçöz, Murat Özdemir

**Affiliations:** 1 Department of General Surgery, Ege University Faculty of Medicine Hospital, İzmir 35100, Türkiye; yigitturk87@gmail.com (Y.T.); bahadir.emre.baki@ege.edu.tr (B.E.B.); gokhan.icoz@ege.edu.tr (G.İ.); 2 Department of Endocrine Surgery, Ozel Saglik Hospital, İzmir 35000, Türkiye; makayozer@yahoo.com; 3 School of Medicine, Aristotle University, 54124 Thessaloniki, Greece; 4 Instituto Português de Oncologia de Coimbra Francisco Gentil, 3000075 Coimbra, Portugal

**Keywords:** atypia of undetermined significance (AUS), fine-needle aspiration biopsy (FNAB), risk of malignancy (ROM), thyroid nodule

## Abstract

*Background and Objectives:* The necessity of repeat fine-needle aspiration biopsy (FNAB) in nodules diagnosed as atypia of undetermined significance (AUS) remains debated. This study evaluates the role of a second FNAB in surgical decision making. *Materials and Methods:* A retrospective analysis was conducted on 131 patients (105 females and 26 males) who underwent surgery following an AUS diagnosis between 2020 and 2024. Patients were grouped based on whether surgery was performed after the first or second FNAB. Demographics, pathology, and ultrasound findings were compared. *Results*: Of the patients, 66 (50.7%) underwent surgery after the first FNAB and 65 (50.3%) after a second AUS diagnosis. Malignancy was detected in 47 out of 66 (71.2%) patients in the single biopsy group and in 44 out of 65 (67.7%) patients in the repeat biopsy group (*p* = 0.804). T1a tumors were more frequent in the first FNAB group (63.8% vs. 37.2%, *p* = 0.021). The EU-TIRADS classifications showed no significant differences. *Conclusions*: The malignancy rate observed (71.2%) exceeds reported rates in the literature, suggesting regional variability. Early-stage cancers were more frequent in patients operated on after the first FNAB, questioning the necessity of repeat biopsy and indicating a potential need to revise current guidelines.

## 1. Introduction

Thyroid nodules are a common medical condition worldwide. Their clinical significance primarily lies in the necessity to rule out malignancy. Although prevalence varies across different populations, approximately 7–15% of thyroid nodules are diagnosed as thyroid cancer [1]. Ultrasonography, which has high sensitivity, is widely used in the diagnosis of thyroid nodules. Most thyroid nodules are benign, and their incidence increases with age and is more common in females [2]. Although ultrasonography is the primary imaging modality used to differentiate between benign and malignant nodules, its interpretation is operator-dependent. To address this, in 2009, the EU-TIRADS (Thyroid Imaging Reporting and Data System) classification was developed to standardize the assessment of malignancy risk based on ultrasound characteristics of thyroid nodules [3]. According to this scoring system, fine-needle aspiration biopsy (FNAB) is recommended for nodules with a high risk of malignancy. To further guide clinical management, the cytological results of FNAB are classified into a six-categorized system ranging from benign to malignant, as proposed by Cibas et al. [4]. Following cytological evaluation, surveillance is recommended for benign lesions (Bethesda categories I–II), whereas surgical intervention is advised for malignant or suspicious lesions (Bethesda categories IV–VI), provided they meet the appropriate clinical criteria [5]. For nodules classified as atypia of undetermined significance or follicular lesion of undetermined significance (AUS/FLUS, Bethesda category III), which fall between the benign and malignant spectrum, clinical management remains less definitive. This category is characterized by the presence of nuclear atypia in the biopsy specimen that is insufficient to be classified into other specific categories [6]. In cases where thyroid ultrasound findings are classified as EU-TIRADS 3 and the biopsy result is reported as AUS, repeat biopsy is recommended [5]. Among all thyroid biopsies, the expected frequency of AUS results is approximately 5–15%. The associated risk of malignancy (ROM), when a non-invasive follicular thyroid neoplasm with papillary-like nuclear features (NIFTP) is not considered malignant, is estimated to range between 10% and 30% [7,8]. When a NIFTP is classified as malignant, the risk of malignancy (ROM) for AUS increases to approximately 40% [9]. Additional molecular and genetic testing approaches have been incorporated into various risk stratification models to further refine cancer risk assessment [10,11]. Due to the limited cost-effectiveness and accessibility of molecular and genetic testing, the primary clinical approach remains aligned with the 2023 European Thyroid Association Clinical Practice Guidelines for the management of thyroid nodules, as endorsed by the American Thyroid Association. These guidelines recommend a repeat biopsy for nodules initially classified as AUS. If the second biopsy also yields an AUS result, surgery is advised, as a malignancy risk of approximately 30% cannot be excluded [5]. In some populations, studies have shown that despite an initial AUS result on FNAB, the final surgical pathology may reveal malignancy in up to 55% of cases [12]. This paper highlights the potential for making a surgical decision without the need for a second biopsy in certain cases. In our study, conducted in Turkey, as an endemic region for thyroid cancer [13], we aim to evaluate the necessity of repeat biopsy in patients whose initial FNAB result was AUS. Additionally, we will assess the malignancy risk in patients who underwent surgery after the first biopsy compared to those who had surgery following a repeat biopsy.

## 2. Methods

This study was designed retrospectively and includes patients over 18 years of age who underwent surgery after receiving an AUS (Bethesda III) result on FNAB between 2020 and 2024 at a tertiary referral center located in Western Turkey. Patients were divided into two groups based on the number of biopsies performed: single biopsy and repeat biopsy groups. Demographic data (such as age and gender), preoperative thyroid ultrasound findings (EU-TIRADS), FNAB results, final postoperative pathology, tumor size, histological subtype, and pathological staging were included in the analysis. Waiting time was defined as the period between the patient’s first surgical evaluation and the subsequent operative intervention. The study protocol was approved by the local Ethics Committee of Medical Research (Approval date: 28 November 2024; Approval number: 24-11.1T/16). For normally distributed variables, independent samples t-tests were used to compare groups. For non-normally distributed paired variables, paired t-tests were applied. For comparisons of non-normally distributed variables between two independent groups, the Mann–Whitney U test was used. Continuous variables were presented as median values with corresponding ranges, while nominal variables were expressed as counts and percentages. Normality was assessed using the Kolmogorov–Smirnov test. The risk of malignancy (ROM) was determined by dividing the total number of histologically confirmed malignant cases by the total number of AUS/FLUS cases diagnosed on FNAB and subsequently resected. Chi-squared tests were conducted to analyze the surgical pathology results of the single biopsy and repeat biopsy groups. A 95% confidence interval was adopted, and a *p*-value of <0.05 was considered statistically significant. All statistical analyses were performed using SPSS software (version 30.0; IBM Corp., Armonk, NY, USA).

## 3. Results

The study included a total of 131 patients, of whom 66 underwent a single biopsy and 65 underwent repeat biopsies. The mean age of the entire cohort was 48.9 ± 12.8 years. There was no significant difference in the mean age between the single biopsy group (50.3 ± 11.3 years) and the repeat biopsy group (47.5 ± 14.2 years) (*p* = 0.211). The gender distribution was similar between groups (*p* = 0.630) (Table 1). Also, the median operation waiting time was comparable between groups: 41 days (IQR: 21.75–81.5) in the single biopsy group versus 43 days (IQR: 23.5–92.5) in the repeat biopsy group (*p* = 0.674).

Regarding EU-TIRADS classification, there were no significant differences between the groups and EU-TIRADS subtypes (*p* = 1000). In the single biopsy group, 1 patient was classified as EU-TIRADS 2, 17 as EU-TIRADS 3, 13 as EU-TIRADS 4, 5 as EU-TIRADS 5, and 30 as not classified (NC), while in the repeat biopsy group, 1 patient was classified as EU-TIRADS 2, 18 as EU-TIRADS 3, 10 as EU-TIRADS 4, 10 as EU-TIRADS 5, and 26 as NC. Among the patients, 56 were categorized as NC due to inconclusive or suboptimal ultrasound imaging quality, which made EU-TIRADS categorization unfeasible. These cases typically lacked sufficient documentation of nodule characteristics such as echogenicity, margins, or calcifications required for EU-TIRADS scoring.

The type of surgical procedure performed (total thyroidectomy vs. lobectomy) was also comparable between groups (*p* = 0.609). A total thyroidectomy was performed in 119 patients, and a lobectomy was performed in 12 patients. Tumor size was significantly larger in the repeat biopsy group, with a median size of 12 mm (IQR: 2.5–21.5) compared to 7 mm (IQR: 2–23) in the single biopsy group (*p* = 0.040). In terms of final pathological diagnosis, papillary thyroid cancer was the most common diagnosis in both groups (45 vs. 41 patients). Other diagnoses included the follicular variant of papillary cancer (18 vs. 21 patients), NIFTP (0 vs. 2 patients), medullary cancer (1 vs. 0), and follicular cancer (2 vs. 1). There were no statistically significant differences in pathological diagnoses between groups (Table 1).

The overall risk of malignancy (ROM) among all patients was 69.5%, with 91 malignant cases identified out of 131 patients. In the single biopsy group, the ROM was 71.2% (47 out of 66 patients), while in the repeat biopsy group, it was 67.7% (44 out of 65 patients). The difference in malignancy rates between the groups was not statistically significant (χ^2^ = 0.06, *p* = 0.804) (Table 2).

Regarding surgical procedures, total thyroidectomy was performed in 90.9% of patients in the single biopsy group and 90.8% in the repeat biopsy group, while lobectomy was performed in 9.1% and 9.2% of patients, respectively.

Histopathological subtypes of malignant tumors included papillary microcarcinoma in 47 patients (mean tumor size 6.4 mm), follicular variant papillary thyroid carcinoma (PTC) in 21 patients (mean size 23.0 mm), classical PTC in 12 patients (mean size 21.1 mm), unusual variants in 5 patients (mean size 30.7 mm), and non-invasive follicular thyroid neoplasm with papillary-like nuclear features (NIFTP) in 2 patients.

The pathological T classification revealed significant differences between the groups. In the single biopsy group, 63.8% of the tumors were classified as T1a, 10.6% as T1b, 19.1% as T2, and 6.4% as T3a. In contrast, in the repeat biopsy group, 37.2% were T1a, 39.5% T1b, 18.6% T2, and 4.7% T3a. The proportion of T1a tumors was significantly higher in the single biopsy group compared to the repeat biopsy group (*p* = 0.021) (Table 3).

## 4. Discussion

While the management of malignant thyroid nodules is well established, there remain areas of uncertainty in the approach to benign-appearing but suspicious nodules. The Bethesda System for Reporting Thyroid Cytopathology provides an effective framework for categorizing thyroid nodules; however, in cases diagnosed as AUS/FLUS, repeat biopsy is still recommended, as the expected risk of malignancy is reported to be around 30% [7]. However, as demonstrated in our study, the observed malignancy risk in clinical practice appears to be higher than previously reported estimates.

Although regional differences exist, several studies in the literature have reported that the risk of malignancy on final surgical pathology following an AUS/FLUS cytology exceeds 30% [14,15,16,17]. In our study, the malignancy risk reached approximately 71.2%, which may be attributed to the fact that Turkey is an endemic region for thyroid cancer [13]. Another potential explanation for the high rate of malignancy among cases initially reported as AUS/FLUS on FNAB may be the tendency of pathologists working at secondary-level centers to provide a more generalized rather than detailed cytological interpretation. This could lead to an increased number of AUS/FLUS classifications. In a study conducted by Yaprak et al. in Turkey involving 108 patients, the postoperative malignancy rate among those initially diagnosed as AUS/FLUS was calculated as 25% [18]. Similarly, in a series of 449 patients reported by Topaloğlu et al., the malignancy rate was found to be 23.4% [19]. In a study conducted at a tertiary care center involving 305 patients who underwent surgery after being diagnosed with AUS/FLUS on FNAB, the malignancy rate was reported to be as high as 38% [20]. According to the national literature, one possible reason for the high malignancy rate observed in our study may be that our center performs surgery on patients referred from secondary-level hospitals who cannot undergo surgery elsewhere and who present with additional risk factors for malignancy, such as vocal cord paralysis or large nodule size. Although an expansion of the study population may lead to a slight decrease in the malignancy rate, the ROM would likely remain higher than the expected range indicated in the Bethesda classification [7].

Xu et al. found a 17% malignancy rate and demonstrated that repeat fine-needle aspiration (FNA) reclassified 72% of cases, supporting its diagnostic value [21]. However, in our setting, where early-stage tumors were more frequent in those operated on after the first biopsy, repeat FNA may delay treatment without significantly lowering the malignancy risk. Of course, in differentiated thyroid cancers, this delay is unlikely to be clinically significant. Similarly, Hathi et al. reported a 13.2–25.3% malignancy rate and highlighted the role of center-specific risk assessment [22]. Given our high malignancy rate, early surgery after the first AUS diagnosis might be more suitable in high-risk populations or referral centers. In contrast, patients who underwent surgery after the initial biopsy were more frequently diagnosed at an earlier pathological T stage. However, given that the early T stage does not significantly impact clinical outcomes in papillary thyroid carcinoma, this finding was not considered clinically meaningful at this stage. In future studies with larger patient cohorts, the inclusion of lymph node status in the analysis could enable a more comprehensive assessment of prognostic staging and may yield more definitive conclusions.

The clinical value of repeat FNAB following an initial AUS/FLUS diagnosis remains a subject of considerable debate. Several recent studies have questioned the utility of repeated FNABs in altering patient management or improving malignancy detection rates in this subgroup of indeterminate thyroid nodules. Jo et al. demonstrated that, among patients with non-diagnostic aspirates, repeating FNAB did not significantly change the malignancy risk, with malignancy rates remaining comparably low after both single and multiple non-diagnostic results (3.5% vs. 6.3%) [23]. Similarly, Evranos et al., in a large cohort of AUS/FLUS nodules, found that the malignancy rates remained remarkably stable across groups undergoing one, two, three, or even four FNABs, with malignancy rates ranging between 27% and 33% regardless of biopsy repetition [24]. Of particular importance, Piticchio et al. reported that only 5.9% of repeat biopsies resulted in a meaningful change in clinical management, and the vast majority of repeated FNABs provided redundant information without altering the therapeutic approach [25]. Our findings align with Piticchio’s study, which questions the clinical utility of repeat FNAB in AUS/FLUS nodules. Only 5.9% of repeated biopsies resulted in management change, emphasizing the limited added value of re-biopsy. Similarly, our data revealed that malignancy rates remained high even after a single AUS result and that repeat FNAB did not significantly improve diagnostic yield or alter management, further supporting early surgical decision making in selected patients. Furthermore, Kahramanca et al. demonstrated that even the timing of repeat FNAB, whether performed early or delayed, had no significant impact on diagnostic adequacy or cytologic outcomes, further questioning the necessity of strict timing protocols for re-biopsy [26]. As a consequence, these findings suggest that routine repeat FNAB following an initial AUS/FLUS diagnosis may have limited incremental value, and that alternative approaches, including early surgical treatment, could reduce patient burden, procedural risks, and healthcare costs.

In addition to oncological considerations, thyroid FNAB procedures impose significant psychological stress, which repeat biopsies may amplify. Şenoymak et al. demonstrated that higher pre-procedural anxiety levels, assessed by the State-Trait Anxiety Inventory (STAI-S), were independently associated with both increased pain perception and non-diagnostic cytology results (OR for non-diagnostic cytology: 3.09 for anxiety and 1.59 for pain) [27]. Similarly, Turkoğlu et al. reported that neck biopsies, including thyroid FNAB, elicited the highest levels of anxiety and stress compared to other anatomical biopsy sites (neck > bone > thorax > abdomen) [28]. Moon et al. further showed that patients awaiting thyroid FNAB had significantly higher anxiety and depression scores than controls, with an increase of nearly five times in anxiety risk (OR 4.97), and that repeated biopsies further exacerbated this psychological burden. These data underscore that repeat biopsies not only delay management but also contribute to increased patient anxiety and procedural distress, supporting the consideration of earlier surgical intervention in selected high-risk populations [29].

To the best of our knowledge, this is the first study to evaluate tumor size in the final pathology of patients who underwent surgery following an AUS/FLUS cytological diagnosis. In our analysis, patients who proceeded to surgery after the first biopsy had a significantly lower median tumor size compared to those who underwent surgery after a repeat biopsy. This finding suggests that the interval between the first and second biopsies may have contributed to tumor growth. One of the limitations of our study is the lack of data on the time interval between the initial and repeat biopsies, which prevents a definitive assessment of whether this duration contributed to the increased tumor size. However, the absence of a significant difference between the two groups in terms of time from diagnosis to surgery suggests that surgical access was similar across both groups. In future studies, documenting the precise interval between the initial and repeat biopsies could help determine whether this delay acts as a risk factor for tumor progression. Furthermore, suppose this interval is shown to contribute to increased tumor size. In that case, it may prompt reconsideration of the current approach and support early surgical intervention in patients with an initial AUS/FLUS result, without waiting for a repeat biopsy. Another limitation of our study is the absence of molecular profiling data, such as BRAF or RAS mutation status of FNAB, which could have enhanced risk stratification and informed surgical decisions. This limitation is primarily due to the financial burden of molecular tests, which cost as much as a surgery. Additionally, as a tertiary referral center, our institution receives complex cases from secondary hospitals, which may lead to selection bias and an overestimation of malignancy risk. These factors should be considered when generalizing our findings to broader populations.

## 5. Conclusions

In conclusion, our findings indicate that the malignancy rate in AUS/FLUS-diagnosed thyroid nodules may be higher than previously estimated, particularly in endemic regions such as Turkey. The absence of a significant difference in overall malignancy rates between the single biopsy and repeat biopsy groups, coupled with the observation of earlier T staging and smaller tumor sizes in the single biopsy cohort, underscores the potential advantage of proceeding with early surgical intervention rather than delaying management for repeat FNAB. Considering both the elevated malignancy risk and the limited incremental diagnostic yield of repeated biopsies, early surgical management may offer a more effective approach in selected patients, while also minimizing diagnostic delays and alleviating the cumulative psychological burden and anxiety often associated with multiple biopsy procedures. These findings emphasize the need to reconsider current management strategies for AUS/FLUS nodules, particularly in high-risk populations.

## Figures and Tables

**Table 1:** Baseline characteristics of patients by biopsy group.

	All Patients (n:131)	Single Biopsy (n:66)	Repeat Biopsy (n:65)	*p* Value
Age (Mean ± SD)	48.9 ± 12.8	50.3 ± 11.3	47.5 ± 14.2	0.211
Gender (F/M)	105/26	54/12	51/14	0.630
Waiting Time Median (IQR)	42 (23–90)	41 (21.75–81.5)	43 (23.5–92.5)	0.674
EU-TIRADS 2	2	1	1	1.000
EU-TIRADS 3	35	17	18	1.000
EU-TIRADS 4	23	13	10	1.000
EU-TIRADS 5	15	5	10	1.000
EU-TIRADS Not Classified	56	30	26	1.000
Operation (Total /Lobectomy)	119/12	60/6	59/6	0.609
Tumor Size Median (IQR)	*10 (4–21.5)*	*7 (2–23)*	*12 (2.5–21.5)*	*0.040*
Main Diagnosis				
*Papillary Thyroid Cancer*	87	45	41	
*Bening*	39	18	21	
*Medullary Thyroid Cancer*	1	1	0	
*Follicular Thyroid Cancer*	3	2	1	
*NIFTP*	2	0	2	

Abbreviation: EU-TIRADS: European Thyroid Imaging Reporting and Data System; F: female; IQR: interquartile range; M: male; NIFTP: non-invasive follicular thyroid neoplasm with papillary-like nuclear features; SD: standard deviation. Statistically significant findings are marked in italics.

**Table 2:** Comparison of risk of malignancy (ROM) between single and repeat biopsy groups.

Biopsy Groups	Malignant/Total Count	Risk of Malignancy (%)	*p* Value
Single Biopsy	47/66	71.2	
Repeat Biopsy	44/65	67.7	0.804

**Table 3:** Comparison of pathological T staging between single and repeat biopsy groups.

Biopsy Groups	T1a	T1b	T2	T3a
Single Biopsy	30 (63.8%)	5 (10.6%)	9 (19.1%)	3 (6.4%)
Repeat Biopsy	16 (37.2%)	17 (39.5%)	8 (18.6%)	2 (4.7%)
Total	46 (51.1%)	22 (24.4%)	17 (18.9%)	5 (5.6%)

## Data Availability

The data presented in this study are available on request from the corresponding author due to ethical reasons.
